# Medical Radar Signal Dataset for Non-Contact Respiration and Heart Rate Measurement

**DOI:** 10.1016/j.dib.2021.107724

**Published:** 2021-12-16

**Authors:** Keisuke Edanami, Guanghao Sun

**Affiliations:** Graduate School of Informatics and Engineering, The University of Electro-Communications, Japan

**Keywords:** Biomedical engineering, Non-contact monitoring, Medical radar, Heart rate, Respiratory rate

## Abstract

Medical radars remotely measure the periodic movements of the chest wall induced by breathing and heartbeat and have been widely recognized in healthcare. To the best of our knowledge, no well-characterized medical radar datasets are shared publicly. Therefore, in this article, we provide non-contact respiratory and cardiac signal datasets measured using a medical radar and simultaneously measured reference signals using electrocardiogram (ECG) and respiratory belt transducer. The datasets were collected from nine healthy subjects using 24.25 GHz and 10.525 GHz Doppler radars at a physiological laboratory in Japan. Furthermore, we generated MATLAB code to pre-process the signals and calculate the respiratory and heart rates. The datasets generated could be reused by biomedical researchers to investigate the signal-processing algorithm for non-contact vital sign measurement.

## Specifications Table

**Table:** 

Subject	Biomedical engineering
Specific subject area	Non-contact biomonitoring; signal processing; clinical application
Type of data	LabVIEW data files (.lvm) & CSV files (.csv) MATLAB code (.m)
How data were acquired	The medical radar signals were acquired using 24.25 GHz (New Japan Radio, NJR4262) and 10.525 GHz (New Japan Radio, NJR4178J) Doppler radars. The ECG and respiratory belt signals were simultaneously acquired using a contact instrument (BIOPAC, BN-REPEC). The analog signals were converted to digital signals using an ADC (USB-6003, National Instruments) and recorded using the LabVIEW data acquisition software.
Data format	Raw
Parameters for data collection	Three channels of non-contact radar signals (Two 24 GHz I/Q channels and one 10 GHz I channel) and contact reference signals (ECG and respiratory signals) were collected for synchronization with the ADC at a sampling rate was 1000 Hz with 16 bits resolution.
Description of data collection	Nine healthy subjects, 5 males and 4 females with an average age of 24±5 were chosen for the experiment, and measurements were conducted on each subject for 10 min. The subjects were instructed to maintain a resting state in a supine position on a bed. The radars were placed under the bed, approximately 15 cm from the subject, to illuminate the heart region. The ECG was attached according to the V5 guidance, and the respiratory belt was placed on the subjects’ abdomen for measurements.
Data source location	Institution: The Graduate School of Informatics and Engineering, The University of Electro-Communications City/Town/Region: Tokyo/Chofu/Chofugaoka 1-5-1 Country: Japan Latitude and longitude: 35.6561°N, 139.5440°E
Data accessibility	Repository name: Mendeley Data Data identification number: 10.17632/6rp6wrd2pr.2 Direct URL to data: https://dx.doi.org/10.17632/6rp6wrd2pr.2

## Value of the Data

• The proposed dataset is essential for the development of non-contact healthcare applications that use medical radars.
• The proposed dataset comprising radar signals from three channels and two reference signals (ECG and respiratory belts) can be reused by biomedical researchers for improving signal processing techniques and to compare machine learning methods using the reference signals.
• The radar signal can also extract physiological parameters such as heart rate variability and respiratory sinus arrhythmia.

## 1 Data Description

### 1.1 The principle of medical radar to non-contact respiration and heart rate measurement

**Fig. 1:**
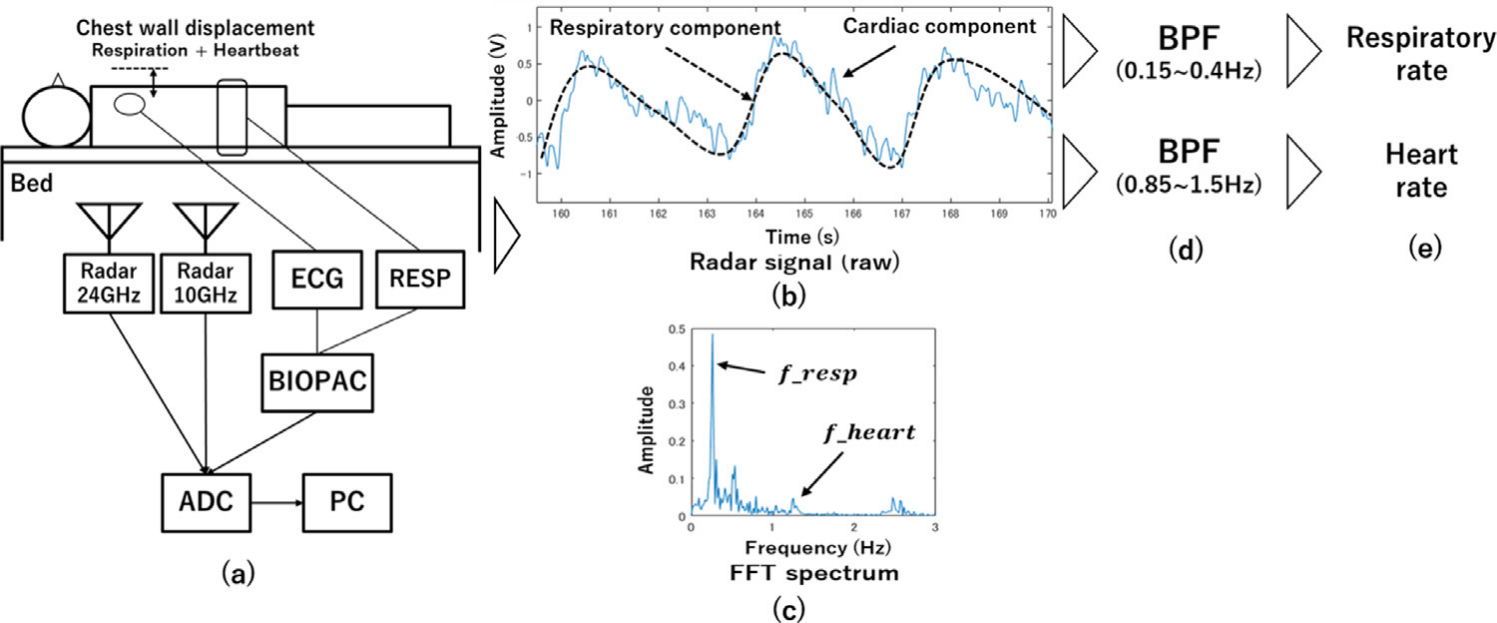
Experiment setting for data acquisition and signal processing for calculating the respiratory and heart rates: (a) Configuration of measurement equipment, (b) Respiratory and cardiac components are captured as the radar raw signal, (c) *f_resp* and *f_heart* are frequencies corresponding to the maximum values of the FFT spectrum, respectively. (d) Extract respective components by BPF (e) HR and RR are calculated from Eqs. (3) and (4)

Medical radars remotely measure periodic movements of the chest wall induced by breathing and heartbeats. The use of medical radars for the non-contact measurement of respiration and heart rate has attracted extensive attention and is expected to have a wide range of healthcare applications, such as sleep apnea detection and monitoring of bedridden patients [1,2]. As seen in Fig. 1, the radar transmitter sends a continuous wave to the body, which is reflected by the surface of the body and received by the receiver. The signal received is mixed with the transmitted signal and output as I and Q signals, as shown in Eqs. (1) and (2), where x(t) is the displacement of the body surface due to respiration and heartbeat, λ is the wavelength, and ϕ is the total residual phase accumulated in the circuit along the transmission path [3].

(1) $$I\left(t\right)=cos\left(\frac{4\pi x\left(t\right)}{\lambda }+\phi \right)$$

(2) $$Q\left(t\right)=sin\left(\frac{4\pi x\left(t\right)}{\lambda }+\phi \right)$$

### 1.2 Signal processing method for calculating respiratory and heart rates

**Fig. 2:**
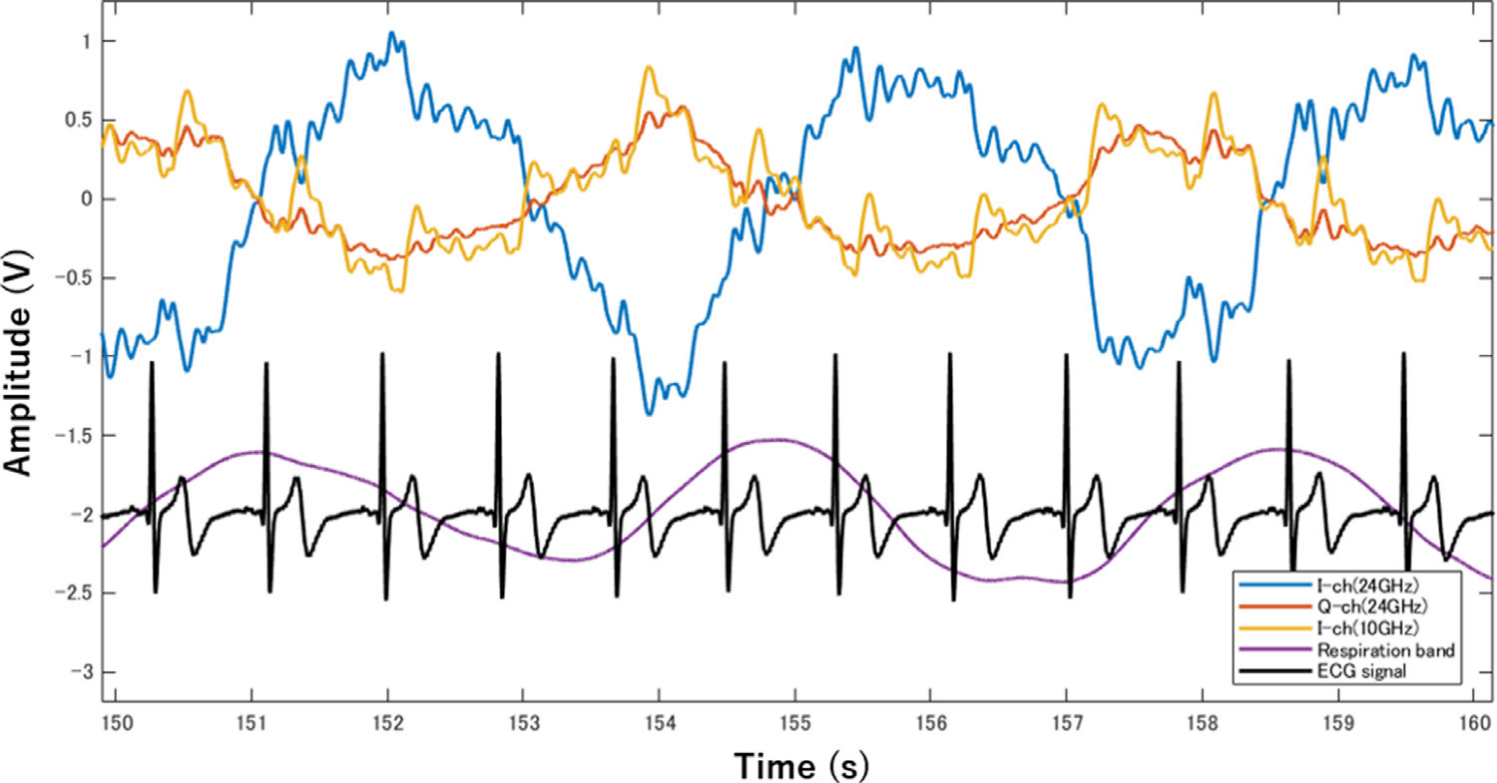
Comparison of waveforms measured from 24 GHz and 10 GHz Doppler radar with reference: It can be seen that the respiratory waveform and radar waveform are synchronized. The peaks in the ECG waveform and the tiny peaks in the radar waveform can also be seen in correspondence.

Fig. 2 shows an example of the measured radar raw signals, reference ECG, and respiratory belt signals. The radar signal comprises a large-amplitude and low-frequency respiratory component and a small-amplitude and high-frequency cardiac component. The fast Fourier transform (FFT) analysis method is commonly used to separate the respiratory and cardiac frequencies. Band-pass filters (BPF) with cut off frequencies of 0.15–0.4 Hz and 0.85–1.5 Hz were used to emphasize the respiratory and cardiac signals, respectively. The respiration rate (RR) per minutes (breaths per minutes, bpm) and heart rate (HR) per minutes (beats per minutes, bpm) were then calculated from the respiration and cardiac signals extracted by the BPF using the conversion Eqs. (3) and (4), where, $f_{resp\;}$and $f_{heart}$ are the frequencies of the respiration and cardiac components, respectively, and are determined from the maximum value of the FFT spectrum as shown in Fig. 1 (c). Table 1 lists the RR and HR values calculated using the MATLAB code, which was also used for pre-processing the signals.

(3) $$\text{RR}=60\;\times f_{resp\;}\;\left[\text{breaths}\;\text{per}\;\text{minutes}\right]$$

(4) $$\text{HR}=60\;\times f_{heart\;}\;\left[\text{beats}\;\text{per}\;\text{minutes}\right]$$

**Table 1:** Respiratory and heart rates calculated by radar and reference signals.

	RR [breaths per minutes]				HR [beats per minutes]			
Subject	24GHz-I	24GHz-Q	10GHz-I	Respiratory belt	24GHz-I	24GHz-Q	10GHz-I	ECG
subject 1	15	15	15	16	62	62	71	74
subject 2	14	15	14	13	72	72	73	74
subject 3	16	16	16	16	71	69	71	71
subject 4	20	19	19	19	63	58	59	70
subject 5	18	18	17	17	61	62	61	61
subject 6	14	14	14	14	58	62	58	83
subject 7	17	17	17	17	56	63	60	69
subject 8	14	17	13	11	55	55	55	54
subject 9	20	20	20	19	56	56	55	55

### 1.3 File structure

The extension of the data file was LVM and CSV. The first 22 lines comprised a header. The variable names were displayed on line 23 in each column. After line 24, the signals were displayed. The information stored in each column was as follows: column 1: time; column 2: 24 GHz radar I-channel; column 3: 24 GHz radar Q-channel; column 4: 10 GHz radar I-channel; column 5: respiratory band signal; and column 6: ECG signal.

## 2 Experimental Design, Materials and Methods

### 2.1 Experimental procedure

Fig. 1 shows the experimental setup. The 24 GHz and 10 GHz radars were placed under the bed to illuminate the area around the subject's heart, with a distance of approximately 15 cm between the subject and the radar. ECG electrodes were placed on the chest according to the V5 guidance. A respiratory belt was attached to the abdomen. The three-channel radar and reference signals were synchronized with the ADC. The experiment was conducted on nine healthy subjects, who were instructed to maintain a supine resting position. The sampling rate was 1000 Hz, and measurements were collected from each subject for 10 min.

### 2.2 Experimental equipment

24GHz Doppler radar: New Japan Radio, NJR4262 (I, Q channels)
10GHz Doppler radar: New Japan Radio, NJR4178J (I channel)
ECG: BIOPAC, BN-REPEC
Respiration: BIOPAC, BN-REPEC
A/D Converter: USB-6003, National Instruments
Software: LabVIEW, National Instruments

### 2.3 Data privacy

All personally identifiable information has been removed from the data files. The radar and reference signals are one-dimensional electrical signals that are highly anonymous.

## Ethics Statement

This study was approved by the Ethics Committee on Human Research of the University of Electro-Communications with the No. 16031.

## CRediT authorship contribution statement

**Keisuke Edanami:** Data curation, Formal analysis, Investigation, Methodology, Software, Validation, Visualization, Writing – original draft. **Guanghao Sun:** Conceptualization, Funding acquisition, Project administration, Resources, Supervision, Writing – review & editing.

## Declaration of Competing Interest

The authors declare no conflicts of interest.

## Appendix Supplementary materials

Applecatin image 1
