# Supplementary figures and images for: Medical Radar Signal Dataset for Non-Contact Respiration and Heart Rate Measurement
Source: Data Brief. 2021 Dec 16;40:107724. doi: 10.1016/j.dib.2021.107724 (PMC8688557; doi:10.1016/j.dib.2021.107724)

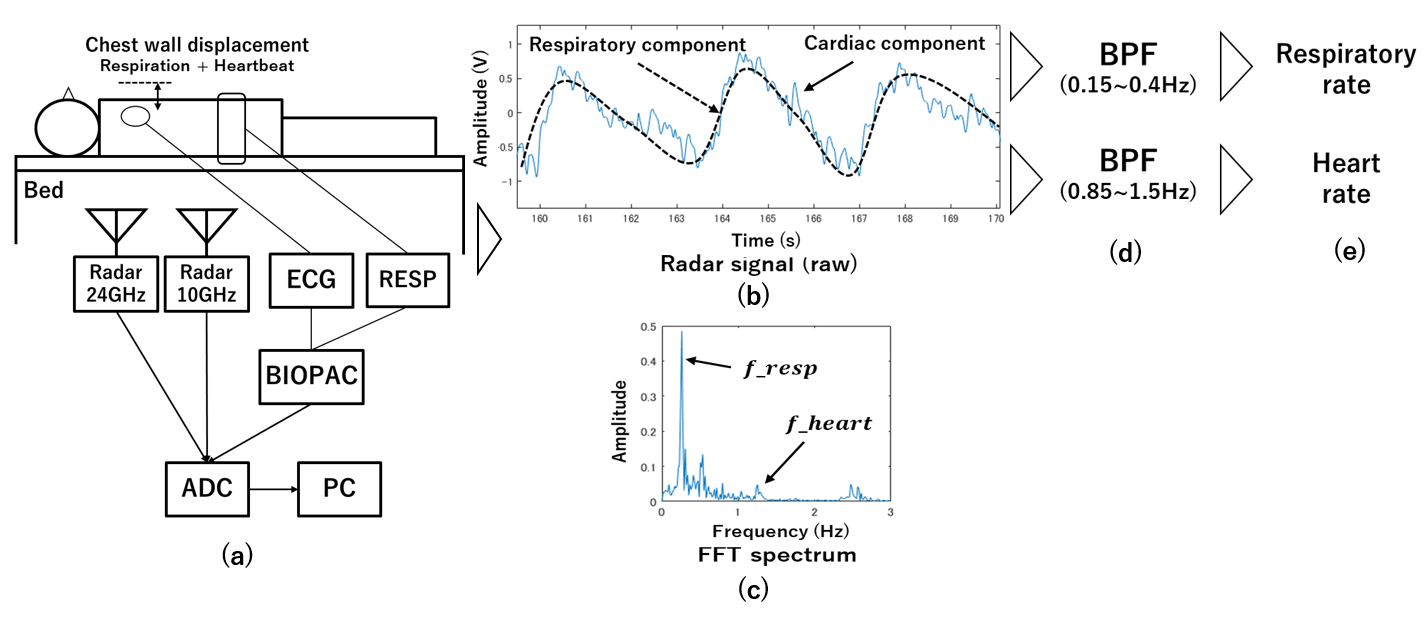

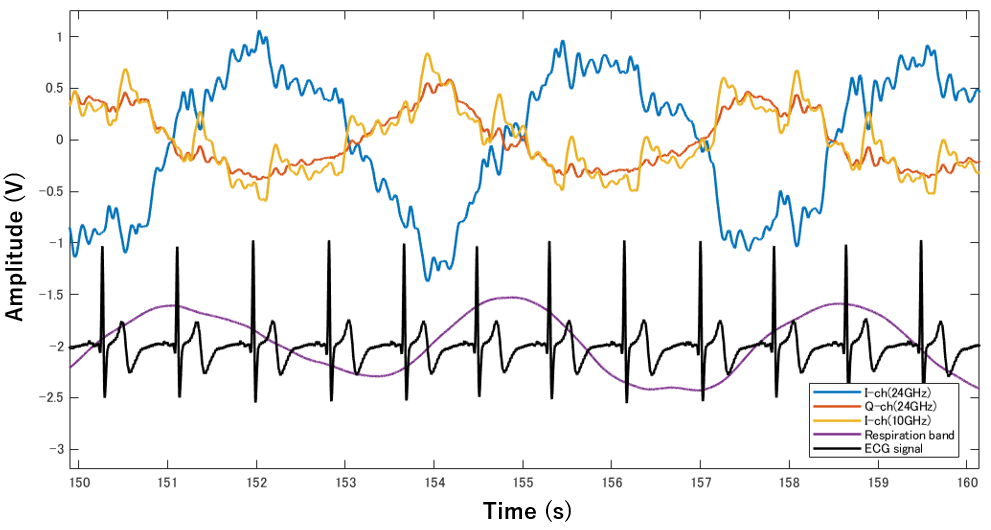

Supplement: Supplementary file 1 [file mmc1.docx]
